# Experimental evidence that primate trichromacy is well suited for detecting primate social colour signals

**DOI:** 10.1098/rspb.2016.2458

**Published:** 2017-06-14

**Authors:** Chihiro Hiramatsu, Amanda D. Melin, William L. Allen, Constance Dubuc, James P. Higham

**Affiliations:** 1 Department of Human Science, Faculty of Design, Kyushu University, 4-9-1 Shiobaru, Minamiku, Fukuoka 815-8540, Japan; 2 Physiological Anthropology Research Center, Kyushu University, 4-9-1 Shiobaru, Minamiku, Fukuoka 815-8540, Japan; 3 Department of Anthropology and Archaeology, University of Calgary, Calgary, Alberta, Canada; 4 Department of Medical Genetics, University of Calgary, Calgary, Alberta, Canada; 5 Alberta Children's Hospital Research Institute, University of Calgary, Calgary, Alberta, Canada; 6 Department of Anthropology, New York University, New York, NY, USA; 7 Department of Biosciences, Swansea University, Swansea, UK; 8 Department of Zoology, University of Cambridge, Cambridge, UK

**Keywords:** colour vision, primate, trichromacy, social signal, face colour variation, reproductive state

## Abstract

Primate trichromatic colour vision has been hypothesized to be well tuned for detecting variation in facial coloration, which could be due to selection on either signal wavelengths or the sensitivities of the photoreceptors themselves. We provide one of the first empirical tests of this idea by asking whether, when compared with other visual systems, the information obtained through primate trichromatic vision confers an improved ability to detect the changes in facial colour that female macaque monkeys exhibit when they are proceptive. We presented pairs of digital images of faces of the same monkey to human observers and asked them to select the proceptive face. We tested images that simulated what would be seen by common catarrhine trichromatic vision, two additional trichromatic conditions and three dichromatic conditions. Performance under conditions of common catarrhine trichromacy, and trichromacy with narrowly separated LM cone pigments (common in female platyrrhines), was better than for evenly spaced trichromacy or for any of the dichromatic conditions. These results suggest that primate trichromatic colour vision confers excellent ability to detect meaningful variation in primate face colour. This is consistent with the hypothesis that social information detection has acted on either primate signal spectral reflectance or photoreceptor spectral tuning, or both.

## 1. Introduction

The selective pressures that led to the evolution of the unique form of trichromatic colour vision in primates from ancestral mammalian dichromacy have been debated for decades [1–4]. One puzzling feature of primate colour vision is that peak sensitivities of photopigments to different wavelengths are not evenly distributed across the visual spectrum, unlike the more regular distribution that is known to occur in many other trichromatic (and tetrachromatic) animals, including bees, hawkmoths, reptiles and passerine birds [5–9]. Cone photopigments in catarrhine primates have peak sensitivities near 420 nm (S—short wavelength, ‘blue’), 530 nm (M—mid wavelength, ‘green’) and 560 nm (L—long wavelength, ‘red’), such that the maximum separation of peak sensitivities between M and L cones is 30 nm, while the S cone is more distantly tuned (110–140 nm away) [10]. While sensitivities of the female platyrrhine LM cones also fall in the range of 530–560 nm [10,11], the separation can be even narrower in a substantial proportion of individuals, depending on the specific combination of polymorphic photopigments. Narrower distribution of long-wavelength-sensitive photopigments leads to a better discrimination of subtle variation in colours in the red or green range at the expense of other hues. Various explanations of the narrow tuning window in primates have been proposed, including non-adaptive ones: (i) the shared origin of primate M and L cones and phylogenetic constraint [12], and/or (ii) a minimizing of the cost to luminance vision via chromatic aberration because M and L signals are pooled to generate a luminance signal [5,13–15].

Alternatively, there are potential adaptive explanations for this narrow tuning. Natural selection may favour enhanced discrimination of wavelengths within the greenish to reddish range of the spectrum at the cost of greater global chromatic discrimination that would be possible with more evenly distributed opsins [5,16]. Two main adaptive hypotheses to explain the evolution of primate trichromacy generally, and the spectral tuning of receptors in particular, have been proposed. A prominent adaptive hypothesis suggests that discriminating among longer-wavelength hues is tuned for the task of finding reddish ripe fruits or young leaves against foliage background (the foraging hypothesis) [2,17,18]. A second adaptive hypothesis proposes that primate trichromacy is tuned to perceive skin colour variation related to changes in blood flow or oxygenation levels, which can provide information about conspecifics' emotional, breeding or health status (the social signal hypothesis) [19]. Indeed, several catarrhine primates exhibit prominent patches of bare skin coloured by blood in the hindquarter, face or chest, and colour changes in these patches act as socio-sexual signals [20–24]. Whether such signalling could explain the evolution and/or maintenance of trichromacy across primates more generally is unclear: large patches of bare skin are less common in platyrrhines, which typically exhibit polymorphic colour vision (both dichromatic and trichromatic individuals in the same population) [11]. An exception is the strikingly red bald uakari [25], although several species show small patches of bare skin, especially around the eyes [19,26]. It is possible that the original tuning of the photoreceptors in primates was related to constraints, or adaptive feeding advantages, with colour signalling benefits arising secondarily and contributing to photoreceptor sensitivity maintenance [27]. Finally, it is also possible that the adaptive significance of trichromacy might be more general, such that detecting ripe fruits and/or skin colour variation are only two among several benefits (general advantage hypothesis) [16,28]. In contrast with the foraging hypothesis [29–32], there has been very little assessment of the social signal hypothesis [24,33,34], although the hypothesis is receiving attention in work on humans [35–37].

In this study, our objective was to test a necessary condition of the hypothesis that the catarrhine trichromatic visual system is well suited for detecting facial skin colour variation in non-human primates. Although this prediction does not distinguish between foraging or social signalling as the original selective pressure influencing the evolution of primate trichromacy and the spectral tuning of the photoreceptors, testing it helps establish whether the social signalling hypothesis merits further investigation. We used an experimental approach to examine whether common primate trichromacy facilitates detection of biologically relevant changes in skin colour in rhesus macaques (*Macaca mulatta*). In females of this species, facial colour variation relates to intra-cycle fertility variation, such that face colour becomes redder and darker around the timing of the peri-ovulatory phase when females are proceptive and fertile [34]. Our objective was to test whether trichromacy increases a viewer's ability to detect variation in reproductive status from rhesus macaque faces by comparing the ability of different simulated visual systems to discriminate proceptive and non-proceptive faces. We also aimed to compare the performance of common catarrhine primate trichromacy, and the trichromacy exhibited by females of many platyrrhine species, with other manipulated forms of colour vision when distinguishing face colour variation. In accordance with the social signal hypothesis, we predicted that observers experiencing trichromatic conditions would show better performance than those experiencing dichromatic conditions, and that unmanipulated common trichromacy would show the best performance among the three forms of trichromacy conditions tested.

Specifically, we presented human participants with images of the faces of proceptive and non-proceptive female rhesus macaques manipulated such that the information available is altered to reflect that available to different colour vision phenotypes. We gave human observers the task of selecting a proceptive female rhesus macaque face between two images of the same monkey, and compared their performance in doing so under six conditions. We tested performance of three forms of trichromacy and three forms of dichromacy. For trichromatic conditions, we tested: (i) an unmanipulated catarrhine vision phenotype (common trichromacy); (ii) a separation between L and M peak sensitivity that was divided by two to simulate the narrower trichromatic colour vision frequently observed in female platyrrhines [11]; and (iii) an evenly distributed spectral separation to simulate a colour vision system with greater global chromatic discrimination. This allowed us to test whether the narrow separation in peak sensitivity between L and M helps trichromatic primates detect biologically relevant skin colour changes. For dichromacy, we simulated conditions in which we removed the common trichromacy sensitivity (iv) to L (protanopia), (v) to M (deuteranopia) or (vi) to S (tritanopia). The former two phenotypes have difficulty distinguishing between colours in the green–yellow–red range of the spectrum, while the latter phenotype has difficulty distinguishing green from blue. This allowed us to further test whether perception of chromatic change is necessary for detecting these facial signals, or whether detection of achromatic changes (e.g. changes in darkness/lightness of the skin) is sufficient.

## 2. Material and methods

### (a) Stimuli

**Figure 1.**
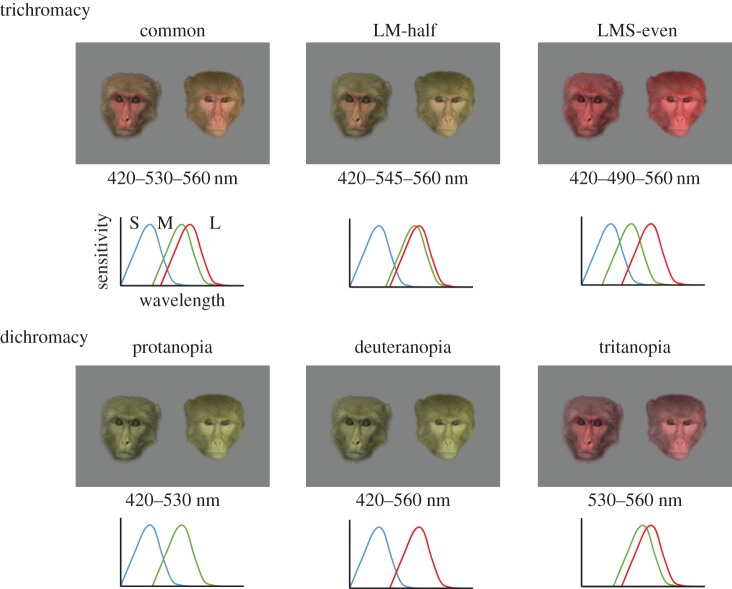
An example of face pair stimuli for common (unmanipulated) trichromacy and simulated colour visions. Numbers below each stimulus indicate the combinations of cone peak wavelength sensitivities (*λ*_max_) used to create the test images. Schematic drawings of cone sensitivities are depicted for each condition.

We used digital photographs of 24 free-ranging adult female rhesus macaques (3.5 years or older) taken on Cayo Santiago, Puerto Rico. The monkey facial area was isolated from the rest of each image for the present experiments. A stimulus image was composed of a pair of faces of the same individual taken outside and during the period of female behavioural proceptivity placed side by side on a uniform grey background (figure 1). Stimulus images were presented on a self-calibrating LCD monitor (EIZO, Color Edge CG277), adjusted to maximal luminance = 80 cd m^−2^, *γ* = 2.2 and white point = 6500 K, in a dark room. In total, 32 face pairs were created, with 16 pairs used as training pairs and the other 16 as test pairs (electronic supplementary material, figure S1). More than one face pair (two or three pairs) was created for seven individuals according to image availability.

### (b) Colour vision conditions

In addition to an unmanipulated vision phenotype (common trichromacy), five other colour vision phenotypes were simulated using custom-written software, colour vision simulator (CVS) [38]. The CVS transforms chromatic information available in images to represent appearance for target colour vision types, which have different cone peak sensitivities (*λ*_max_) from the common type or that lack certain cone types. Using the *λ*_max_ values of the source (a common trichromat observer) and target phenotype, the CVS generates shifted photopigment sensitivity curves and performs a pixel-by-pixel colour transformation by shifting the hue and saturation coordinates from the colour space of a common trichromat to that of the desired phenotype [38]. We assumed sensitivities of catarrhine L, M and S cones peak at 560 (L), 530 (M) and 420 nm (S). We simulated three dichromatic conditions—protanopia, deuteranopia and tritanopia, which lack L, M or S cones, respectively. We further simulated two putative trichromatic conditions—trichromacy with a narrow LM spectral separation (half of the common type) with peak sensitivities of the M and L cones at 545 and 560 nm (LM-half trichromacy), and trichromacy with peak sensitivity of the M cone half-way between the S and L peaks at 490 nm (LMS-even trichromacy) (figure 1). The LM-half trichromacy was intended to simulate the narrower trichromatic colour vision frequently observed in platyrrhines [11].

### (c) Participants

Sixty participants (30 women and 30 men) ranging from 19 to 41 years old (mean age = 23.2, s.d. = 4.3) with normal acuity and normal (common trichromatic) colour vision as diagnosed by an anomaloscope completed the experiment. Participants were randomly assigned into one of six conditions, with 10 (5 women, 5 men) individuals assigned to each condition.

### (d) Procedures

Participants were verbally instructed that during the experiment, they would need to select as quickly as possible which of the two face images of a given stimulus pair was collected during the proceptive period, when females are seeking reproductive opportunities (two-alternative forced choice). No *a priori* information about face colour in the proceptive state was given. The experiment was split into two parts: training and test trials. In training trials, positive feedback was given when participants selected a proceptive face, and negative feedback if a non-proceptive face was selected. In this way, participants had the opportunity to learn to attend to features of proceptive faces. In test trials, positive feedback was always given, irrespective of correctness, so that participants could not further learn critical aspects of signals from test pairs.

Each participant conducted six sessions that consisted of one training session and five subsequent test sessions. The training session consisted of 32 training trials based on 16 training pairs of faces replicated to counterbalance the location (left or right) of a proceptive face. Each test session consisted of 64 trials in total, with 32 training trials as above and 32 test trials based on 16 test pairs of faces (here again duplicated to counterbalance the location). We repeated the test session five times to examine if any learning effects appear as sessions proceeded. The test trials examined whether participants could generalize the important aspects of proceptive faces learned during the training trials to test pairs. The training trials in the test sessions served to keep up the motivation of participants and to give further chance to learn features of proceptive faces by giving positive and negative feedback according to accuracy. The order of trials was randomized within each session. After all sessions were completed, participants were asked to report which cues they relied on during the task.

### (e) Analysis

To examine the effect of colour vision type, the effect of learning through sessions, the effect of trial type (training or test) and the possible effect of sex, while controlling for participant identity and stimulus image identity, we analysed all responses from 60 participants with generalized linear mixed models (GLMMs) using the package lme4 in R statistical analysis software (R v. 3.4.0). First, we analysed if the correct (proceptive) image was selected in a GLMM model using a binomial distribution with a logit link function. In this model, accuracy in each trial was the response variable, colour vision condition, session, trial type, sex, interactions between colour vision and other factors and an interaction between session and trial type were fixed effects, and participant IDs blocked by stimulus image identity (face pair combinations) were random effects. Maximum-likelihood with Laplace approximation was used for the GLMM fitting. Type III Wald *χ*^2^ tests were used to examine the significance of each fixed effect specified in the model. Differences between colour vision conditions and between women and men in each colour vision condition were analysed by post hoc least-squares-means multiple-comparison tests with the Tukey method for adjusted *p*-values. The reaction time (RT) in each trial was similarly analysed with linear mixed models (LMMs) after log transformation of positively skewed raw data. Restricted maximum-likelihood (REML) was used for LMM fitting. The RT (log scale) of each trial was the response variable, colour vision condition, session, trial type, accuracy, sex and all interactions were fixed effects, and participant IDs blocked by stimulus image identity were again random effects.

In primates, the neural circuitry that extracts the redness signal compares the outputs between M and L cone photoreceptors; the neural circuitry that extracts lightness (achromatic) signal is derived from the sum of outputs of M and L cones [5]. For common trichromacy and tritanopia, whose redness and lightness signals can be calculated from the output of common type M and L photoreceptors, we conducted partial correlation analyses to examine the relative contribution of redness and lightness differences between paired faces on accuracy and RT while controlling for the influence of the other factor (redness or lightness). For each face pair, we averaged the mean accuracy and RT across all participants in each condition during all trials. Redness and lightness were calculated as (L − M)/(L + M) and (L + M)/2, respectively, where M and L indicate input to each cone transformed from the camera's RGB values [39] and averaged across a portion of a face [40]. Redness and lightness differences between faces in each pair were calculated by subtracting the value of a non-proceptive face from that of a proceptive face. This yields positive redness difference and negative lightness difference if the proceptive face is redder and darker. We also conducted the Pearson correlation analyses between lightness differences and mean accuracy or RT for protanope and deuteranope, whose lightness output was solely through the M or L cone, respectively. The *α*-level was set to 0.05 for all analyses. The frequency of reported cues that participants relied on during the experiment was summarized for each colour vision condition based on debriefing after the experiment. For more details on the material and methods, see the electronic supplementary material.

## 3. Results

### (a) Effects of colour vision on accuracy

**Figure 2.**
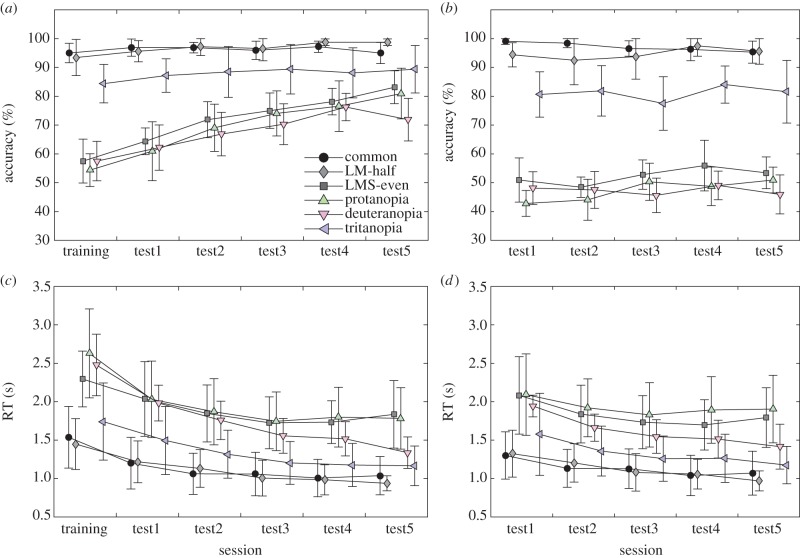
Transition of percentage accuracy and RT in each condition. The change of mean accuracy across participants through sessions for (*a*) training pairs and for (*b*) test pairs. The change of mean RT across participants through sessions for (*c*) training pairs and for (*d*) test pairs. Note that test pairs were not included in the training session. Error bars indicate 95% CI of the means, truncated at 100% for the accuracy variable. The sample size included in each plot is 10 individuals. Circle, common trichromacy; diamond, LM-half trichromacy; square, LMS-even trichromacy; triangle, protanopia; inverted triangle, deuteranopia; arrowhead, tritanopia. (Online version in colour.)

Differences in performance among colour vision conditions for training and test trials were reflected in accuracy, RTs and learning effects (figure 2). There were significant fixed effects of colour vision, session and trial type on accuracy, and significant interactions between colour vision and other factors, and between session and trial type (all *p* < 0.0001 except for trial type: *p* = 0.0053, type III test; table 1). Post hoc multiple comparisons of least-squares means showed that the accuracy of common (catarrhine) and LM-half (platyrrhine) trichromatic conditions was significantly higher than other conditions, but they were not different from each other. Furthermore, the accuracy of tritanopia was significantly higher than protanopia, deuteranopia and LMS-even trichromatic conditions (all adjusted *p* < 0.05; electronic supplementary material, table S1).

**Table 1.** Analysis of deviance table in Type III Wald *χ*^2^ tests of the GLMM for accuracy and the LMM for RT.

accuracy				reaction time			
effect	*χ*^2^	d.f.	Pr > *χ*^2^	effect	*χ*^2^	d.f.	Pr > *χ*^2^
(intercept)	234.12	1	<0.0001	(intercept)	1.68	1	0.19
colour vision	394.44	5	<0.0001	colour vision	213.03	5	<0.0001
session	15.90	1	<0.0001	session	128.64	1	<0.0001
trial type	7.77	1	0.0053	trial type	3.21	1	0.073
sex	0.0032	1	0.95	accuracy	7.57	1	0.0059
colour vision : session	51.97	5	<0.0001	sex	164.39	1	<0.0001
colour vision : trial type	29.20	5	<0.0001	colour vision : session	164.49	5	<0.0001
colour vision : sex	48.56	5	<0.0001	colour vision : trial type	2.02	5	0.85
session : trial type	54.05	1	<0.0001	colour vision : accuracy	38.98	5	<0.0001
				colour vision : sex	190.16	5	<0.0001
				session : trial type	63.03	1	<0.0001
				session : accuracy	15.72	1	<0.0001
				trial type : accuracy	2.50	1	0.11

### (b) Effects of colour vision on reaction time

There were significant fixed effects of colour vision, session, accuracy and sex on RT (all *p* < 0.0001 except for accuracy: *p* = 0.0059; table 1). Interactions between colour vision and session, between colour vision and accuracy, between colour vision and sex, between session and trial type, and between session and accuracy showed significant effects (all *p* < 0.0001; table 1). Post hoc multiple comparison tests showed that RT was significantly faster for common and LM-half trichromatic conditions than for all other conditions and that tritanopia was significantly faster than protanopia, deuteranopia and LMS-even trichromatic conditions (all adjusted *p* < 0.05; electronic supplementary material, table S1). There was no significant difference between common and LM-half trichromacies, or among protanopia, deuteranopia and LMS-even trichromacy, except that deuteranopia was significantly faster than protanopia (*p* < 0.05; electronic supplementary material, table S1). A post hoc comparison showed that RT was significantly faster in women than in men (*p* < 0.05). However, multi-model comparisons using the Akaike information criterion (AIC) and likelihood ratio tests suggested that colour vision has a much stronger effect on performance than sex (electronic supplementary material, table S2).

### (c) Chromatic versus achromatic signal use and participant feedback

There were significant positive partial correlations between redness differences and accuracy for both common trichromacy (partial *r* = 0.56, *p* = 0.029) and tritanopia (partial *r* = 0.83, *p* < 0.001) for training trials when lightness was controlled. Similarly, significant negative partial correlations between redness difference and RT were observed in common trichromacy for training trials (partial *r* = −0.80, *p* < 0.001), but only for tritanopia for both training (partial *r* = −0.82, *p* < 0.001) and test trials (partial *r* = −0.57, *p* = 0.026). For lightness, common trichromacy showed a significant negative partial correlation with accuracy (partial *r* = −0.66, *p* = 0.008) when redness was controlled, but a positive partial correlation with RT (partial *r* = 0.68, *p* = 0.005). There were no significant correlations between lightness difference and accuracy or RT in either protanopia or deuteranopia (table 2; electronic supplementary material, figure S2). All participants who experienced common trichromacy, LM-half trichromacy and tritanopia reported that they relied on the redness of faces to determine proceptivity. Full details on participant feedback are provided in electronic supplementary material, table S3.

**Table 2.** Relative contribution of redness and lightness difference to accuracy and RT. Partial correlation coefficients (partial *r*) in common trichromacy and tritanopia and correlation coefficients (*r*) in protanopia and deuteranopia are shown. Asterisks indicate *p*-values of significant correlations.

colour vision condition	trial type	accuracy		reaction time	
		redness difference	lightness difference	redness difference	lightness difference
common trichromacy	training	0.56*	−0.66**	−0.80***	0.68**
	test	0.20	−0.12	−0.34	0.19
tritanopia	training	0.83***	0.44	−0.82***	−0.08
	test	0.40	−0.20	−0.57*	0.08
protanopia	training		0.22		0.12
	test		0.21		−0.19
deuteranopia	training		−0.09		0.30
	test		−0.20		−0.25

*p < 0.05, **p < 0.01, ***p < 0.001.

## 4. Discussion

We used a functional substitution approach [38,41,42] to examine whether primate trichromatic colour vision characterized by narrow sensitivity between L and M wavelength is well suited for detecting naturally occurring colour variation in female rhesus macaque faces. Our results indicate that the separation of cones is an important factor for discriminating face colour variation in the context of detecting reproductive status of female rhesus macaques. As predicted, common catarrhine and LM-half (platyrrhine) trichromacy enabled higher accuracy and faster identification (lower RTs) when compared with other simulated colour vision types, suggesting that having L and M cones with less than or equal to 30 nm separation enables receivers to extract biologically important colour variation in facial signals. This type of tuning between photoreceptors and signals has been described in a taxonomically wide range of species, including for example in *Photuris* and *Photinus* fireflies [43,44], and in *Heliconius* butterflies [45].

The lack of significant differences between performance under common and LM-half trichromatic conditions is interesting because in platyrrhines, which have a sex-linked multi-allelic opsin gene, many trichromatic females possess narrowly separated LM cones which can be as little as 7–17 nm [11,46]. Our results suggest that both of these phenotypes enable the extraction of meaningful skin colour variation, although it is known that other various factors, including olfactory, haptic, behavioural and auditory signals, may also play important roles in sexual behaviour in female platyrrhines [47–49]. The improvement of accuracy through sessions for training pairs in protanopia, deuteranopia and LMS-even trichromacy (figure 2*a*), and the lack of this effect for test pairs (figure 2*b*), may reflect participant learning the correct images of training faces according to feedback in the absence of other reliable clues.

Interestingly, RT of women was faster than men (electronic supplementary material, table S1 and figure S3). There were also interactions between colour vision and sex (table 1), with significantly higher accuracy in women under the LM-half trichromatic condition where red–green colour signals are available but less salient compared with the common trichromatic condition (electronic supplementary material, table S1). These results may indicate a potential advantage for women in detecting relatively weak redness signals [50]. However, an opposite trend is observed in accuracy under the tritanopia condition, where men showed significantly higher accuracy than women (electronic supplementary material, table S1). Investigating the underlying cause of this sex-biased difference is an interesting topic for the future. It is important to mention that due to the number of model parameters and our sample size, there is potential for model overfitting. Consequently, we ran simpler models without interaction terms and confirmed that the main effects of colour vision persisted. Additionally, we ran separate analyses for women and men, and demonstrate that the effect of colour vision is the robust factor influencing performance (electronic supplementary material, table S4). These analyses also showed similar differences among colour vision conditions as shown in electronic supplementary material, table S1 (electronic supplementary material, table S5).

Our experiments suggest that the spectral tuning of the photopigments possessed by catarrhine primates is advantageous for the detection of social signals. This is consistent with a necessary condition of the social signal hypothesis, which posits that photoreceptor sensitivities have been selected for their ability to detect such signals. However, our experiments do not assess whether fitness benefits due to social signalling may have been involved in the original selective pressures acting on photoreceptor sensitivities. One possibility is that these sensitivities are related to evolutionary constraints, or to adaptive fitness benefits in other domains such as feeding advantages, with fitness benefits related to social signalling subsequently being involved in their maintenance. For example, frugivory is hypothesized to have played a key role in shaping primate cognitive and sensory evolution [51], and may have been an important initial selective pressure favouring primate trichromacy [3,27,29]. Further, our experiment does not distinguish between selection acting on the sensitivity of photoreceptors versus the predominant wavelengths of signals. Indeed, given the need for photoreceptors to be used in a wide range of tasks, and given the plasticity of signal expression, this latter scenario is perhaps a likely explanation for our results. Comparative analysis of signal and photoreceptor evolution across diverse primate taxa may help to distinguish these alternatives. For instance, chromatic changes around sexual skin during the reproductive period are observed even in dichromatic lemurs [52] and platyrrhines with polymorphic colour vision [53]. The functionality of these chromatic changes as social signals in those species awaits further study.

In addition to spectral tuning of the photoreceptors, contribution of redness and lightness signals to performance is another aspect that characterizes visual systems with different colour vision. Partial correlation analyses revealed that both redness and lightness differences between faces contributed to the performance of common trichromacy for training pairs. All participants who experienced common and LM-half trichromatic conditions reported that they used facial redness as a cue, the former reports being consistent with the correlation analyses. Reflecting this use of redness cues, individuals performed better in tritanopia compared with LMS-even trichromacy and with the other dichromatic conditions. Partial correlation analyses also show that lightness differences correlated with the performance of participants under common trichromacy, although this was not reported by participants. Therefore, the difference in debriefing terminology used may reflect how humans describe colour (i.e. a tendency to report categorical colours such as red [54] instead of reporting the nuance of colours in terms of their chromatic and achromatic elements, despite the fact that lightness differences also contributed to the performance). The absence of significant correlations between redness or lightness differences and performance in test trials in common trichromacy is probably due to a ceiling effect of performance once observers are experienced or due to lower colour variation in the test pairs of faces (see electronic supplementary material, figure S2).

Although it might be desirable to conduct experiments in which monkeys are the test subjects as well as the stimuli (because post-receptor processing is not necessarily the same in the humans and monkeys), our functional substitution approach has many benefits over experiments using monkeys. These include increased communicative abilities of humans, ease of repeating experiments, the availability and standardization of subjects, and the ability to control the conditions under which images are observed. While primate experiments are feasible, they are subject to more variation in the conditions under which stimuli are viewed and in the underlying state of the subject, and there are limitations on sample sizes, especially when undertaking multiple conditions, as in the present study. It should be noted, however, that our results were obtained under fairly ideal conditions: simultaneous presentation, constant lighting, pose and viewing distance, against a grey background. Monkeys in the wild must assess face colour signals under varying lighting, pose and distances, and perhaps even remember how the current face compares with others viewed previously. Consistent with this, a prior study has suggested a role of familiarity for improving male performance in assessing the colours exhibited by females [55].

## 5. Conclusion

Overall, our results suggest that common trichromacy gives individuals a clear benefit in exploiting the variation in skin coloration associated with important aspects of individual condition, such as intra-cycle variation in female fertility. To our knowledge, this is the first empirical support that a necessary condition of social signal hypothesis is met, indicating that the relative spectral positioning of the M and L photoreceptors in catarrhine trichromatic visual system is well suited for detecting facial skin colour variation in non-human primates. However, our results do not allow assessment of whether this is a primary or a secondary function of primate routine trichromacy. Indeed, an alternative explanation is that routine trichromacy became fixed early on in catarrhine evolution, due to fitness benefits from improved foraging ability, and that subsequently the red–green colour channel became co-opted for socio-sexual signalling [27]. Given the plasticity of signals and the need for photoreceptors to fulfil many tasks, it is highly plausible that selection has acted primarily on primate signal wavelengths rather than the photoreceptors themselves. Further studies of the proximate and ultimate factors influencing primate colour vision are needed to investigate these different scenarios.

## Supplementary Material

ESM_Primate trichromacy and colour signals

## Data accessibility

The dataset supporting this article is uploaded in Dryad Digital Repository (http://dx.doi.org/10.5061/dryad.6q3db) [56].
