# Supplementary material for: Experimental evidence that primate trichromacy is well suited for detecting primate social colour signals
Source: Proc Biol Sci. 2017 Jun 14;284(1856):20162458. doi: 10.1098/rspb.2016.2458 (PMC5474062; doi:10.1098/rspb.2016.2458)
Supplement: ESM_Primate trichromacy and colour signals [file rspb20162458supp1.pdf]

Experimental evidence that primate trichromacy is well suited for detecting primate social colour signals

Electronic Supplementary Material

Chihiro Hiramatsu, Amanda D. Melin, William L. Allen, Constance Dubuc and James P. Higham

Supplementary Material and Methods

(a) Stimuli

We used digital photographs of 24 free-ranging adult female rhesus macaques (3.5 years or older) taken on Cayo Santiago, Puerto Rico. Details of image collection have been described elsewhere [1, 2]. Briefly, images were taken outside and during the period of female behavioural proceptivity at the peak of the mating season 2012 (March-May) with a characterised digital camera (Canon EOS Rebel T2i). Female behavioural proceptivity was established using all male-female socio-sexual interactions collected on a daily basis as part of a project on female reproductive strategies. Females were considered proceptive if they actively solicited mating activity or mating, or if they were observed with a vaginal plug (see Dubuc et al. (2012) [3]). White balancing and exposure adjustment were undertaken for each monkey image by photographing a colour standard (X-rite ColorChecker Passport) under as similar an illumination as possible immediately after. The monkey facial area in images of each female was isolated using Photoshop CS5 (Adobe Systems). For the present experiments, a stimulus image was composed of a pair of faces taken of the same individual during proceptive and a non-proceptive phases, and paired faces were placed side by side on an uniform (24cd/m^2) grey background (figure 1). In total, 32 face pairs were created, with 16 pairs used as training pairs and the other 16 as test pairs (figure S1). One pair was created for 17 females, two pairs were created for 6 females and three pairs were created for one female according to the availability of photographs. Stimulus images was presented on a hardware calibrated LCD monitor (EIZO, Color Edge CG277) with sRGB colour mode at 1920×1200 pixels. The calibration of the monitor was undertaken automatically with the built-in self-calibration sensor and adjusted to maximal luminance = 80 cd/m^2, γ = 2.2 and white point = 6500 K.

(b) Colour vision conditions

In addition to an unmanipulated vision phenotype (common trichromacy), five other colour vision phenotypes were simulated using custom-written software, Colour Vision Simulator (CVS), developed for functional substitution approaches to colour vision [4]. The CVS transforms images to

represent appearance for target colour vision types, which have different cone peak sensitivities from the common type or that lack certain cone types. The main processing of the CVS is a pixel-by-pixel colour transformation by shifting the hue and saturation coordinates from the colour space of an observer's colour vision to a simulated colour vision. First, the user specifies the λ_{max} values of the source (usually, common human trichromatic colour vision) and target (simulated colour vision) phenotype. Using those values, the program generates shifted photopigment sensitivity curves according to function 1, a 4th order Gaussian function,

$$f(x,n) = \sum_{i=1}^{4} \left(a_i e^{\frac{(\frac{x-n}{s} - b_i)^2}{2c_i}} \right) \qquad \text{function 1}$$

where n is the peak sensitivity of the photopigment, s is a polynomial function ($s = 1.626^{-05}n^2 - 0.01319n + 3.343$), and for the *i*th occurrence, the values of a, b and c are adjusted according to the values in Table S5. Curves generated by this function best fit the overall shape of photopigment sensitivity curves derived from the perceptual 10° cone fundamental data for which the effects of macular and lens filtering were removed [5-8]. Each photopigment sensitivity curve generated has the same shape. Idealized cone fundamentals are then recreated for the source and target phenotypes using the generated sensitivity curves and the difference between the generated photopigment curves and the 10° human cone fundamentals (i.e. a composite pre-receptoral filter). The program assumes that the different photopigment types contribute equally to the perception of a white light source, and normalizes the response curves so that the sensitivity at each wavelength is expressed as a proportion of the maximum sensitivity for the relevant receptor type. Then the program creates a colour space table for the source and target phenotypes that lists the relative sensitivities of the component photopigments to each wavelength in 1 nm increments, using the idealized cone fundamentals, and based on standard daylight (D65) illumination. Intermediate values are derived from the table via linear interpolation. For each image in the trial, the original RGB values for each pixel are converted to chromaticity and luminance values. The chromaticity values are located in the source colour space table to find the predominant hue; the saturation value is defined as the relative distance of the chromaticity value from the white point. To find the modified chromaticity values, the hue value is then located in the target colour space table, and the target saturation value is projected from the white point. An inverse transformation to the new RGB colour space creates the task stimulus. The luminance values are held constant during this transformation process [4]. Melin et al. (2013) used this program to conduct a search task with images that simulate dichromatic monkey visions and showed that the performance of simulated conditions was comparable to that of congenitally red-green colour-deficient human observers who saw unmanipulated images [4].

We assumed sensitivities of catarrhine L, M and S cones peak at 560 (L), 530 (M) and 420 nm (S), which is common for catarrhines, including both macaques and humans [9-11]. We also simulated three dichromatic conditions – protanopia, deuteranopia and tritanopia, which lack L, M or S cones, respectively. We further simulated two putative trichromatic conditions – trichromacy with a narrow LM spectral separation (half of the common type) with peak sensitivities of the M and L cones at 545 and 560 nm (LM-half trichromacy), and trichromacy with peak sensitivity of the M cone half-way between the S and L peaks at 490 nm (LMS-even trichromacy) (figure 1). The LM-half trichromacy was intended to simulate the narrower trichromatic colour vision frequently observed in New World monkeys [12]. The absence of two differing inputs to the luminance channel in protanopia and deuteranopia was expected to improve the ability to detect changes in the achromatic signal [13].

(c) Participants

Sixty participants, 30 women (F), ranging from 19 - 41 years old (mean age = 22.4, *SD* = 4.4) and 30 men (M), ranging from 20 - 39 years old (mean age = 23.9, *SD* = 4.2) mainly students and staff of Kyushu University, completed the experiment. All participants were diagnosed as common trichromats as determined by an anomaloscope (Neitz OT- Ⅱ). All participants reported that they had greater than normal acuity (20/20) with uncorrected or corrected vision and could read letters that were presented at approximately one degree of visual angle from 60 cm. There were no participants with strabismus. Participants were randomly assigned into one of six conditions with 10 (5 women, 5 men) individuals assigned to each condition.

(d) Procedures

Participants sat c.a. 60 cm from the display with a chin rest in a dark room and were verbally instructed in Japanese that one of the face pairs from the same individual was in a proceptive status (seeking reproductive opportunities) and the other was in non-proceptive status and to select the former by a mouse click (two-alternative forced choice) precisely and quickly within 10 sec. No *a priori* information about face colour in the proceptive state was given. Prior to the main experiment, participants were given 4 practice trials in order to familiarise themselves with the trial processes and begin to learn the appearance of proceptive faces. The images used in practice trials were not used in subsequent training and test trials, and results of practice trials were not analysed. The participants were then given a training session that consisted of only training trials to learn the difference between proceptive and non-proceptive faces, followed by five test sessions that consisted of both training and test trials. The training session consisted of 32 training trials using 16 training pairs by

replicating each face pair twice to valance the location (left or right) of a proceptive face. In test sessions 1-5, test trials using 16 test pairs were introduced to examine whether participants could generalize the important signals of proceptive faces learned during the training session to new test pairs. Each session consisted of 32 training trials and 32 test trials with all stimulus pairs by duplicating each pair to valance the location.

In practice and training trials, positive feedback (a high pitched sound and a green circle around a face) was given when participants selected a proceptive face, and negative feedback (a low pitched sound and red circle around a face) if a non-proceptive face was selected. In this way, participants had the opportunity to learn to attend to features of proceptive faces, and feedback on training trials in test sessions 1-5 also maintained performance and motivation. In test trials positive feedback was always given irrespective of correctness so that participants could not further learn the critical signals from test pairs. Participants were not told there were two differing types of recorded trials and were not able to distinguish test trials from training trials. Every trial started with instruction text to select a proceptive face for 2 sec. Following the instruction text, one pair of face images was automatically presented for up to 10 sec, or until a participant responded. Trials were automatically continued until a participant finished the 32 trials in the training session and 64 trials in the session 1-5, they then took a sufficient break (1~5 min) depending on the fatigue of participants before beginning the next session. After all sessions were completed, participants were asked to report their strategy (i.e. which cues they relied on) during the task. Multiple cues were allowed to be reported.

(e) Analysis

To examine the effect of colour vision condition, the effect of learning through sessions, and the effect of trial type (training or test) and sex (women or men), while controlling for participant identity and stimulus image identity, we analysed 21120 responses from 60 participants with generalized linear mixed models (GLMMs) using the package lme4 in R statistical analysis software (R version 3.4.0). First, we analysed if the correct (proceptive) image was selected (1: correct or 0: incorrect) in a GLMM model with a binomial distribution with logit link function, where accuracy in each trial was the response variable, and colour vision condition, session, trial type, sex, interactions between colour vision and other factors and an interaction between session and trial type were fixed effects, and participant IDs blocked by stimulus image identity (face pair combinations) were random effects. Maximum likelihood with Laplace approximation was used for the GLMM fitting. Type III Wald chi-square tests were used to examine the significance of each fixed effect specified in the model with the package car in R. Post hoc multiple comparisons of least square means were performed with the Tukey method for adjusted p-values by the package lsmeans in R. Reaction time

(RT) of each trial was similarly analysed in linear mixed models (LMMs) after log transformation of positively skewed raw data. Restricted maximum likelihood (REML) was used for LMM fitting. The RT (log scale) of each trial was the response variable, colour vision condition, session, trial type and accuracy (correct or incorrect), sex, interactions between colour vision and one of other factors and interactions between other factors were fixed effects, and participant IDs blocked by stimulus image identity were again random effects. We also examined the models without interaction terms to see if the conclusions are robust with fewer predictors.

To investigate the relative importance of colour vision and sex we compared models using Akaike information criterion (AIC) scores. We also checked that Bayesian information criterion (BIC) scores and model deviances showed a consistent pattern. Likelihood ratio tests were also performed to compare the full model and the models without sex or colour vision. We further examined the models with data from only women or men separately to see if the conclusions of colour vision effects were robust irrespective of sex differences. All GLMM and LMM analyses were performed with the package lme4 in R software and raw data and codes are provided in Dryad Digital Repository: http://dx.doi.org/10.5061/dryad.6q3db.

For common trichromacy and tritanopia, whose redness and lightness signals can be calculated from the output of common type M and L photoreceptors, we conducted partial correlation analyses to examine the relative contribution of redness and lightness differences between paired faces on accuracy (percentage of correct responses among all responses) and RT while controlling for the influence of the other factor (redness or lightness). For each face pair, we averaged the mean accuracy and RT across all participants in each condition during all trials. Redness and lightness were calculated as (L-M)/(L+M) and (L+M)/2, respectively, where M and L indicate input to each cone transformed from the camera's RGB values according to the method described in Stevens et al. (2007) [14] and averaged across a portion of a face [1]. Redness and lightness differences between faces in each pair were calculated by subtracting the value of a non-proceptive face from that of a proceptive face. This yields positive redness difference and negative lightness difference if the proceptive face is redder and darker. We also conducted Pearson correlation analyses between lightness differences and mean accuracy or RT for protanope and deuteranope, whose lightness output was solely through the M or L cone, respectively. We omitted putative LM-half and LMS-even conditions from this analysis since only M and L outputs calculated from "common" sensitivities were available. All correlation analyses were conducted in MATLAB programing environment (Mathworks). The alpha-level was set to 0.05 for all analyses. The frequency of reported cues that participants relied on during the experiment were summarized for each colour vision condition based on debriefing after the experiment.

Supplementary Results

Sex differences

Interestingly, sex effects were observed in our data. There was a significant main effect of colour vision in RT and a significant interaction between colour vision and sex in both accuracy and in RT (full model, results of fixed effects shown in table 1).

However, excluding colour vision effects from the full model increased AIC scores dramatically (accuracy ΔAIC = 1176, RT ΔAIC = 1332), whereas excluding sex effects made a much smaller, though still important change to model performance (accuracy ΔAIC = 40, RT ΔAIC = 388) (table S2). Likelihood ratio tests showed that excluding the colour vision and its interaction terms from the full model significantly decrease the fitness of the model compared to excluding the sex and its interaction terms for accuracy ($\chi^2 = 1163.3$, df = 14, $p < 0.0001$) and for RT ($\chi^2 = 892.1$, df = 19, $p < 0.0001$). These results indicate that the effect of colour vision is significantly larger than that of sex, demonstrating that while sex is an important factor on performance, colour vision is the primary factor driving variation in performance.

Supplementary Discussion

Our finding of sex differences in performance, while not the focus of the current investigation, merits interpretation and further investigation. Differences between males and females are a common finding in psychophysical studies, including those investigating colour perception [15]. Identifying the underlying mechanism is difficult. It is possible that results are a consequence of differences in motivation, attention, cognition or colour vision perception. While colour vision defects are male-linked, participants were screened for having normal colour vision. Nonetheless there may be a female advantage for making fine colour vision discriminations especially around red-green regions in the colour space [16, 17] as female advantage in accuracy was largest in the LM-half trichromatic condition where red-green colour signals are available but less abundant compared to the common trichromatic condition (table S1). However, our face stimuli contain complex information and the task demands meant we are unable to isolate whether female advantage is due to difference in colour perception. Further research is necessary to uncover the underlying reason behind the observed sex effect.

References

1. Dubuc C, Allen WL, Maestripieri D, Higham JP. 2014 Is male rhesus macaque red color ornamentation attractive to females? *Behav. Ecol. Sociobiol.* **68**, 1215-1224. (doi:10.1007/s00265-014-1732-9)

2. Dubuc C, Winters S, Allen WL, Brent LJN, Cascio J, Maestripieri D, Ruiz-Lambides AV, Widdig A, Higham JP. 2014 Sexually selected skin colour is heritable and related to fecundity in a non-human primate. *Proc. R. Soc. B* **281**, 20141602. (doi:10.1098/Rspb.2014.1602)

3. Dubuc C, Muniz L, Heistermann M, Widdig A, Engelhardt A. 2012 Do males time their mate-guarding effort with the fertile phase in order to secure fertilisation in Cayo Santiago rhesus macaques? *Horm. Behav*. **61**, 696-705. (doi:doi.org/10.1016/j.yhbeh.2012.03.003)

4. Melin AD, Kline DW, Hickey CM, Fedigan LM. 2013 Food search through the eyes of a monkey: A functional substitution approach for assessing the ecology of primate color vision. *Vision. Res.* **86**, 87-96. (doi:10.1016/j.visres.2013.04.013)

5. Bone RA, Landrum JT, Cains A. 1992 Optical density spectra of the macular pigment in vivo and in vitro. *Vision Res.* **32**, 105–110. (doi:10.1016/0042-6989(92)90118-3)

6. Stiles, WS, Burch, JM. 1959. Npl colour-matching investigation: Final report. *Optica Acta*. **6**, 1–26. (doi:doi.org/10.1080/713826267)

7. Stockman A, Sharpe LT. 2000 The spectral sensitivities of the middle- and long- wavelength sensitive cones derived from measurements in observers of known genotype. *Vision Res.* **40**, 1711–1737. (doi:doi.org/10.1016/S0042-6989(00)00021-3)

8. Stockman A, Sharpe LT, Fach CC. 1999 The spectral sensitivity of the human short-wavelength cones derived from thresholds and color matches. *Vision Res.* **39**, 2901–2927. (doi:doi.org/10.1016/S0042-6989(98)00225-9)

9. Baylor DA, Nunn BJ, Schnapf JL. 1987 Spectral sensitivity of cones of the monkey *Macaca fascicularis*. *J. Physiol.* **390**, 145-160. (doi:10.1113/jphysiol.1987.sp016691)

10. Schnapf JL, Kraft TW, Baylor DA. 1987 Spectral sensitivity of human cone photoreceptors. *Nature* **325**, 439-441. (doi:10.1038/325439a0)

11. Jacobs GH, Deegan JF. 1999 Uniformity of colour vision in Old World monkeys. *Proc. R. Soc. B* **266**, 2023-2028. (doi:10.1098/rspb.1999.0881)

12. Jacobs GH. 2007 New World monkeys and color. *Int. J. Primatol.* **28**, 729-759. (doi:10.1007/s10764-007-9168-y)

13. Morgan MJ, Adam A, Mollon JD. 1992 Dichromats detect colour-camouflaged objects that are not detected by trichromats. *Proc. R. Soc. B* **248**, 291-295. (doi:10.1098/rspb.1992.0074)

14. Stevens M, Parraga CA, Cuthill IC, Partridge JC, Troscianko TS. 2007 Using digital photography to study animal coloration. *Biol. J. Linn. Soc.* **90**, 211-237. (doi:10.1111/j.1095-8312.2007.00725.x)

15. Geary DC. 2010 *Male, female: The evolution of human sex differences* (2nd ed.). Washington, DC: American Psychological Association.

16. Bimler DL, Kirkland J, Jameson KA. 2003 Quantifying variations in personal color spaces: Are there sex differences in color vision? *Color Res. Appl.* **29**, 128–134. (doi: 10.1002/col.10232)

17. Murray IJ, Parry NRA, McKeefry DJ, Panorgias A. 2012 Sex-related differences in peripheral human color vision: A color matching study. *J. Vision* **12**:18. (doi: 10.1167/12.1.18)

Supplementary figures and tables

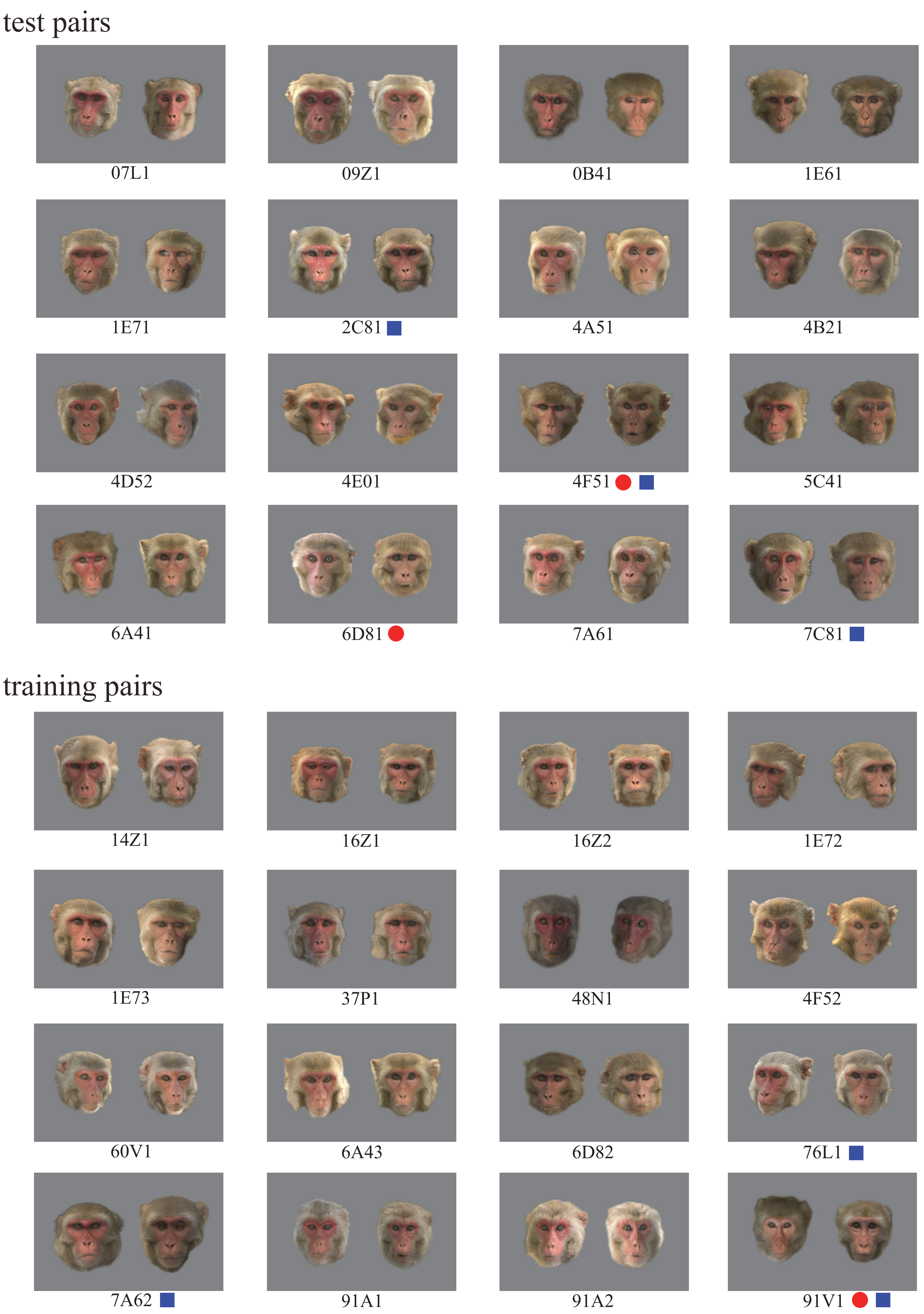

Figure S1. All face pairs used in the study.
16 pairs were used as training pairs and another 16 pairs were used as test pairs. Proceptive faces are placed at left side in this figure but location was counter-balanced in the experiment. IDs of pairs are indicated below each pair. Proceptive faces were usually both redder and darker than non-proceptive faces. Red circles beside IDs indicate a non-proceptive face that is redder than the proceptive faces, while blue squares indicate a non-proceptive face that is darker than the proceptive face.

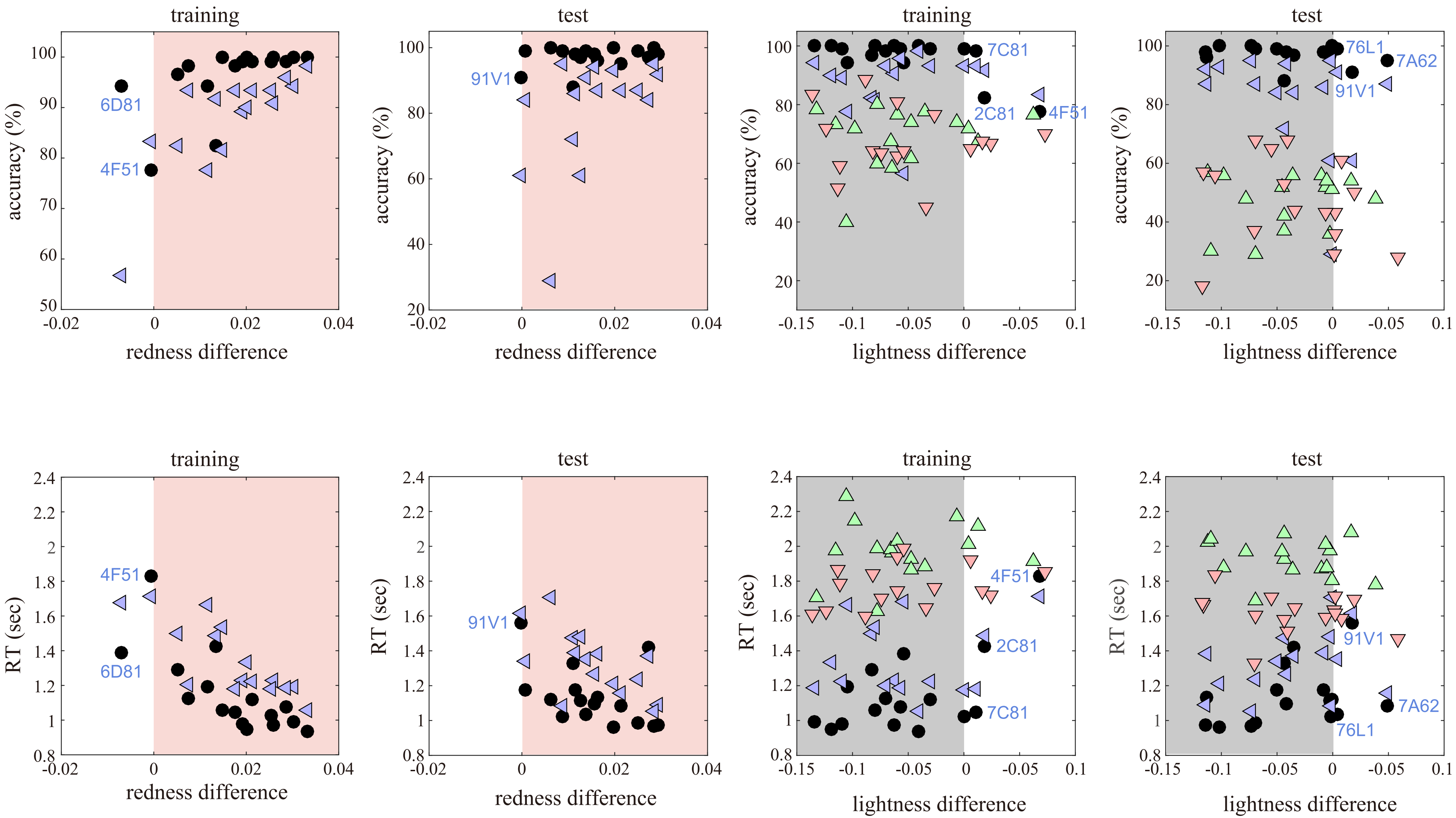

Figure S2. Scatter plots between redness or lightness differences and accuracy or RT.
Each point indicates the mean accuracy or RT for one of 16 training pairs or 16 test pairs averaged across 10 participants. IDs beside black circles (performance of common trichromacy) indicate face pairs whose differences in redness or lightness is opposite to the normal pattern for normal trichromacy (see figure S1). Note that the range of y-axes (accuracy) varies across panels. Partial correlation coefficients, partial *r*, or correlation coefficients, *r*, between two factors in each condition are shown in table 2. Black circle: common trichromacy, blue arrowhead: tritanopia, green triangle: protanopia, pink inverted triangle: deuteranopia. Red and grey areas indicate the normal pattern where the proceptive-face is redder or darker, respectively.

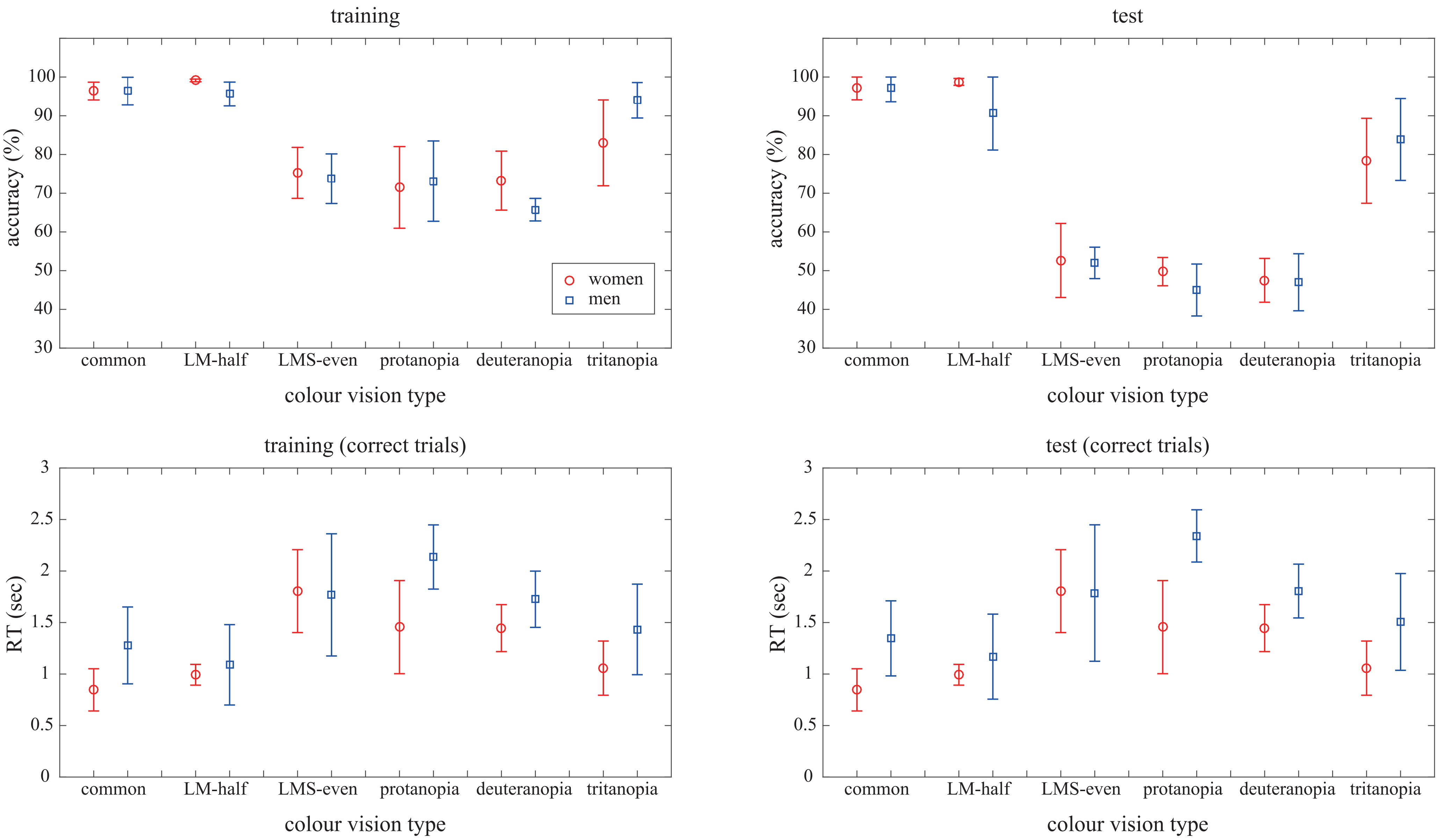

Figure S3. Sex difference on accuracy and reaction time of correct trials.
Accuracy and reaction time (RT) under each colour vision condition are presented separately for women and men. Accuracy of training pairs (upper left), accuracy of test pairs (upper right), RT of training pairs in correct trials (lower left) and RT of test pairs in correct trials (lower right). Each plot indicates mean accuracy or RT ± 95% CI (truncated at 100% for accuracy). Sample size of each plot is 5 individuals of each sex.
Red circle: women, cyan square: men. Note that only correct trials are included in the figure for RT to remove the large fluctuation of response speed in incorrect trials, which probably reflects the individual differences in strategy (very slow response or rapid guesswork) in difficult trials.

Table S1. Results of multiple comparisons between colour vision conditions and comparisons between women and men under each colour vision condition for accuracy and RT.

differences of least squares means adjusted for multiple comparisons									
accuracy					RT				
colour vision contrast	estimate	SE	z.ratio	p.value	colour vision contrast	estimate	SE	z.ratio	p.value
C - D	4.09	0.19	21.61	<.0001	C - D	-0.41	0.02	-18.3	<.0001
C - E	3.82	0.19	20.12	<.0001	C - E	-0.48	0.02	-21.41	<.0001
C - H	-0.07	0.23	-0.31	1	C - H	-0.02	0.02	-0.75	0.98
C - P	4.07	0.19	21.44	<.0001	C - P	-0.51	0.02	-22.42	<.0001
C - T	2.00	0.19	10.36	<.0001	C - T	-0.14	0.02	-6.08	<.0001
D - E	-0.28	0.14	-1.92	0.39	D - E	-0.07	0.02	-3.2	0.018
D - H	-4.16	0.20	-20.56	<.0001	D - H	0.40	0.02	17.72	<.0001
D - P	-0.02	0.14	-0.16	1	D - P	-0.09	0.02	-4.29	0.0003
D - T	-2.09	0.15	-13.52	<.0001	D - T	0.28	0.02	12.85	<.0001
E - H	-3.89	0.20	-19.18	<.0001	E - H	0.47	0.02	20.86	<.0001
E - P	0.25	0.14	1.77	0.49	E - P	-0.02	0.02	-1.11	0.88
E - T	-1.82	0.16	-11.70	<.0001	E - T	0.35	0.02	16.09	<.0001
H - P	4.14	0.20	20.41	<.0001	H - P	-0.49	0.02	-21.88	<.0001
H - T	2.07	0.21	10.04	<.0001	H - T	-0.12	0.02	-5.36	<.0001
P - T	-2.07	0.16	-13.34	<.0001	P - T	0.37	0.02	17.16	<.0001
women - men contrast in each colour vision	estimate	SE	z.ratio	p.value	women - men contrast in each colour vision	estimate	SE	z.ratio	p.value
C, F - C, M	-0.02	0.30	-0.06	1	C, F - C, M	-0.39	0.03	-12.82	<.0001
D, F - D, M	0.13	0.20	0.65	1	D, F - D, M	-0.17	0.03	-5.70	<.0001
E, F - E, M	0.03	0.20	0.13	1	E, F - E, M	0.1	0.03	3.25	0.052
H, F - H, M	1.85	0.33	5.60	<.0001	H, F - H, M	-0.06	0.03	-2.15	0.59
P, F - P, M	0.14	0.20	0.70	1	P, F - P, M	-0.34	0.03	-11.29	<.0001
T, F - T, M	-0.94	0.23	-4.10	0.0025	T, F - T, M	-0.3	0.03	-10.05	<.0001

C: common trichromacy, D: deuteranopia, E: LMS-even trichromacy, H: LM-half trichromacy, P: protanopia, T: tritanopia,
F: female (women), M: male (men). For colour vision contrast, results are averaged over the levels of: trial type, sex.
For women - men contrast in each colour vision, results are averaged over the levels of trial type.
Results for accuracy are given on the log odds ratio (not the response) scale.
Results for RT are given on the log (not the response) scale.
P value adjustment: Tukey method.

Table S2. Comparison of models with/without colour vision or sex effect.

	model	AIC	BIC	deviance (-2logL)
accuracy	full	16213	16420	16161
	without sex	16253	16413	16213
	without colour vision	17389	17434	17377
RT	full	15881	16159	15811
	without sex	16269	16500	16211
	without colour vision	17213	17203	17103

Table S3. Reported cues and strategies during interview after the experiment.

colour vision condition		reported cues and strategies
trichromacy	common	redness of face colour (10, forehead:1, eyes:1), facial (eye) expression (3), memory (2), deepness of face colour (1), lightness of face colour (1), saturation of face colour (1), shape of eyes (1), sharpness of pictures (1)
	LM-half	redness of face colour (10, eyes:2), facial (eye) expression (2), memory (2), shape of eyes (2), length between nose and mouth (1), lightness of face colour (1)
	LMS-even	gaze (3), memory (3), shape of eyes and mouth (3), brightness (shineness) of face and fur (3), facial expression (2), redness of face colour (2), pupil size (2), saturtation of face colour (1)
dichromacy	protanopia	shape of face parts (7), facial expression (4), lightness of face colour (4), pupil size (4), memory (3), gaze (2), fur texture (1), sharpness of pictures (1)
	deuteranopia	facial expression (4), memory (4), shape of face, eyes and mouth (4), length between nose and mouth (2), lightness of face colour (2), pupil size (2), wrinkles around eyes (2), colour around eyes (1), darkness of face colour (1)
	tritanopia	redness of face colour (10, eyes:1), colour (brightness, darkness) of eyes (3), facial expression (3), saturation of redness (2), gaze (1), lightness of face colour (1), memory (1)

Numbers in parentheses indicate the number of individuals who reported each cue or strategy.
For face parts, numbers in parentheses indicate the frequency at which participants reported focusing on different parts of the face. Multiple cues were allowed to be reported.

Table S4. Analysis of deviance table for Type III Wald chi-square tests for the simple model and models with data from only women or men.

accuracy							
		simple model		women		men	
effect	d.f.	X^2	Pr > X^2	X^2	Pr > X^2	X^2	Pr > X^2
(intercept)	1	426.33	<.0001	148.67	<.0001	131.66	<.0001
colour vision	5	952.30	<.0001	249.4	<.0001	223.94	<.0001
session	1	164.10	<.0001	15.87	<.0001	2.56	0.11
trial type	1	100.15	<.0001	2.97	0.085	4.76	0.029
sex	1	0.64	0.42	-	-	-	-
	-	-	-	-	-	-	-
colour vision : session	5	-	-	39.66	<.0001	23.22	<.0001
colour vision : trial type	5	-	-	18.35	0.0025	13.85	0.017
	-	-	-	-	-	-	-
session : trial type	1	-	-	19.32	<.0001	36.10	<.0001
	-	-	-	-	-	-	-

RT							
		simple model		women		men	
effect	d.f.	X^2	Pr > X^2	X^2	Pr > X^2	X^2	Pr > X^2
(intercept)	1	50.42	<.0001	0.02	0.90	55.59	<.0001
colour vision	5	1123.01	<.0001	221.58	<.0001	46.48	<.0001
session	1	2223.92	<.0001	60.61	<.0001	63.34	<.0001
trial type	1	0.28	0.60	1.65	0.20	1.83	0.18
sex	1	228.99	<.0001	-	-	-	-
accuracy	1	16.43	<.0001	1.67	0.20	5.24	0.022
colour vision : session	5	-	-	98.96	<.0001	182.62	<.0001
colour vision : trial type	5	-	-	0.46	1	2.03	0.84
colour vision : accuracy	5	-	-	4.71	0.15	36.76	<.0001
session : trial type	1	-	-	45.31	<.0001	21.6	<.0001
session : accuracy	1	-	-	6.75	0.0094	6.5	0.011
trial type : accuracy	1	-	-	0.08	0.77	4.42	0.035

Table S5. Results of multiple comparisons between colour vision conditions for accuracy and RT under the simple model without interaction terms and under the models with data from only women or men.

differences of least squares means adjusted for multiple comparisons												
accuracy												
model	simple model				women				men			
colour vision contrast	estimate	SE	z.ratio	p.value	estimate	SE	z.ratio	p.value	estimate	SE	z.ratio	p.value
C - D	4.11	0.19	21.58	<.0001	4.01	0.26	15.19	<.0001	4.22	0.28	15.32	<.0001
C - E	3.85	0.19	20.17	<.0001	3.79	0.26	14.33	<.0001	3.89	0.28	14.11	<.0001
C - H	0.2	0.21	0.95	0.93	-1.03	0.37	-2.77	0.062	0.87	0.29	2.95	0.038
C - P	4.1	0.19	21.48	<.0001	3.99	0.26	15.09	<.0001	4.2	0.28	15.19	<.0001
C - T	2.03	0.19	10.5	<.0001	2.48	0.27	9.36	<.0001	1.53	0.28	5.37	<.0001
D - E	-0.27	0.15	-1.82	0.45	-0.22	0.2	-1.12	0.88	-0.33	0.21	-1.58	0.61
D - H	-3.91	0.19	-21.04	<.0001	-5.04	0.34	-14.81	<.0001	-3.35	0.25	-13.47	<.0001
D - P	-0.01	0.14	-0.08	1	-0.02	0.19	-0.11	1	-0.02	0.21	-0.08	1
D - T	-2.08	0.16	-13.29	<.0001	-1.52	0.2	-7.48	<.0001	-2.69	0.24	-11.45	<.0001
E - H	-3.65	0.19	-19.59	<.0001	-4.82	0.34	-14.16	<.0001	-3.02	0.25	-12.13	<.0001
E - P	0.25	0.15	1.75	0.50	0.2	0.2	1	0.92	0.31	0.21	1.5	0.67
E - T	-1.82	0.16	-11.56	<.0001	-1.31	0.2	-6.38	<.0001	-2.36	0.24	-10.02	<.0001
H - P	3.9	0.19	20.94	<.0001	5.02	0.34	14.74	<.0001	3.33	0.25	13.35	<.0001
H - T	1.83	0.19	9.68	<.0001	3.52	0.34	10.32	<.0001	0.66	0.26	2.53	0.12
P - T	-2.07	0.16	-13.2	<.0001	-1.5	0.2	-7.37	<.0001	-2.67	0.24	-11.32	<.0001
RT												
model	simple model				women				men			
colour vision contrast	estimate	SE	z.ratio	p.value	estimate	SE	z.ratio	p.value	estimate	SE	z.ratio	p.value
C - D	-0.41	0.02	-18.34	<.0001	-0.53	0.03	-17.84	<.0001	-0.3	0.03	-8.79	<.0001
C - E	-0.49	0.02	-21.74	<.0001	-0.73	0.03	-24.76	<.0001	-0.23	0.03	-6.89	<.0001
C - H	0	0.02	0.04	1	-0.17	0.03	-5.10	<.0001	0.15	0.03	4.31	0.0002
C - P	-0.51	0.02	-22.79	<.0001	-0.54	0.03	-18.19	<.0001	-0.48	0.03	-13.91	<.0001
C - T	-0.14	0.02	-6.31	<.0001	-0.19	0.03	-6.31	<.0001	-0.09	0.03	-2.74	0.067
D - E	-0.08	0.02	-3.39	0.0091	-0.2	0.03	-7.18	<.0001	0.07	0.03	2.01	0.33
D - H	0.41	0.02	18.39	<.0001	0.36	0.03	11.31	<.0001	0.45	0.03	13.57	<.0001
D - P	-0.1	0.02	-4.47	0.0001	-0.01	0.03	-0.35	1	-0.18	0.03	-5.36	<.0001
D - T	0.27	0.02	12.11	<.0001	0.34	0.03	12.13	<.0001	0.21	0.03	6.32	<.0001
E - H	0.49	0.02	21.79	<.0001	0.56	0.03	17.73	<.0001	0.38	0.03	11.62	<.0001
E - P	-0.02	0.02	-1.08	0.89	0.19	0.03	6.83	<.0001	-0.24	0.03	-7.39	<.0001
E - T	0.35	0.02	15.51	<.0001	0.55	0.03	19.37	<.0001	0.14	0.03	4.32	0.0002
H - P	-0.51	0.02	-22.84	<.0001	-0.37	0.03	-11.63	<.0001	-0.62	0.03	-18.87	<.0001
H - T	-0.14	0.02	-6.36	<.0001	-0.02	0.03	-0.56	0.99	-0.24	0.03	-7.33	<.0001
P - T	0.37	0.02	16.57	<.0001	0.35	0.03	12.49	<.0001	0.38	0.03	11.67	<.0001

C: common trichromacy, D: deuteranopia, E: LMS-even trichromacy, H: LM-half trichromacy, P: protanopia, T: tritanopia.
For colour vision contrast, results are averaged over the levels of: trial type.
Results for accuracy are given on the log odds ratio (not the response) scale.
Results for RT are given on the log (not the response) scale.
P value adjustment: Tukey method.

Table S6. The values used by the Colour Vision Simulator to generate photopigment sensitivity curves.

value of *i*	a	b	c
1	0.94077	-5.02	67.39
2	0.01053	19.48	6.499
3	0.26324	-246.92	152.4
4	0.21105	47.88	38.52
